# Predictors of tuberculosis treatment outcome at Senkatana clinic in Lesotho

**DOI:** 10.11604/pamj.2024.49.91.41882

**Published:** 2024-11-26

**Authors:** Richard Mwamba Kabuya, Alfred Musekiwa, Simbarashe Takuva, Lehana Thabane, Lawrence Mbuagbaw

**Affiliations:** 1 Senkatana Antiretroviral Therapy Clinic, Maseru, Lesotho,; 2 School of Health Systems and Public Health, Faculty of Health Sciences, University of Pretoria, Pretoria, South Africa,; 3 Department of Health Research Methods, Evidence and Impact, McMaster University, Hamilton, Ontario, Canada,; 4 Biostatistics Unit, Father Sean O´Sullivan Research Centre, St Joseph´s Healthcare Hamilton, Hamilton, Ontario, Canada

**Keywords:** Predictors, tuberculosis, treatment outcomes, Lesotho

## Abstract

**Introduction:**

tuberculosis (TB) is one of the top ten causes of death and the leading cause from a single infectious agent called Mycobacterium tuberculosis. This study aims to evaluate TB treatment outcomes among patients on first-line anti-tuberculosis treatment and identify the factors associated with successful TB treatment outcomes at Senkatana TB clinic in Lesotho from 2015-2017.

**Methods:**

a registry-based retrospective cohort study of all TB first-line cases recorded from 2015 to 2017 was conducted at Senkatana TB clinic. Data were captured and cleaned in Epi info version 7, and exported into Stata version 14 for analysis. Bivariate logistic regression analysis was used to determine factors associated with TB treatment outcome with p-value <0.05 indicating statistical significance.

**Results:**

a total of 1,027 TB patients were registered between 2015 and 2017. Of these, 602 (58.6%) were males and 425 (41.4%) were females, with a mean age of 39 years (SD ±12.5). A total of 843 (82.1%) patients were co-infected with HIV, of which 92.3% (n=778) were on anti-retroviral therapy (ART). The analysis of HIV co-infected with TB patients by age showed that the TB/HIV co-infection rate varies with age (p<0.001). Overall treatment success rate was at 73.4% (n= 754) and 273 (26.6%) had poor treatment outcomes, and 118 (11.5%) patients died. The odds of successful TB treatment outcome were higher in females than males (78.1% vs 70.1%, OR 1.52, 95% CI: 1.14 - 2.03, p=0.004). With regards to age, the odds of successful TB treatment outcome were higher for the 20-24 years age group (88.2% vs 65.3%, OR 3.98, 95% CI: 1.42 - 11.22, P=0.009) and 55-59 years (91.7% vs 65.3%, OR 5.84, 95% CI: 1.56 - 21.88, P=0.009), compared to ≥ 65 years age group. In addition, successful TB treatment outcomes were higher among HIV co-infected TB patients who were taking ART during TB treatment than those not taking ART (75.8% vs 23.8%, OR 11.70, 95% CI: 6.40 - 21.43, P<0.001). Patients observed by family members or friends were more likely to develop treatment success (aOR: 1.87, 95% CI: 1.13 - 3.08). Factory workers in high-risk groups had successful treatment outcomes (aOR: 1.77, 95% CI: 1.04 - 3.01).

**Conclusion:**

tuberculosis treatment success rate was low and constant over the period of three years. Death rate, loss to follow, and not evaluated were high among our study participants and above the World Health Organization (WHO) target. In unadjusted analyses, female sex, younger age, HIV co-infected taking ART, having a treatment observer, and belonging to high-risk groups, were significantly associated with successful TB treatment outcome.

## Introduction

According to the World Health Organization (WHO), Global Tuberculosis Report 2019, around 10 million people are infected with tuberculosis (TB) each year. Tuberculosis is one of the top 10 causes of death, and the leading cause from a single infectious agent (*Mycobacterium tuberculosis*), ranking above HIV/AIDS [1]. Tuberculosis and Human Immunodeficiency Virus (HIV) are diseases of public health concern for countries with limited resources. The intricate linkage of TB and HIV infection for nearly the past 3 decades poses a major threat to the international community´s effort to achieve the health-related United Nations Sustainable Development Goals for TB and HIV infection [2]. Lesotho is one of the 15 countries with the highest per capita case incidence of 632/100,000 (WHO Global Tuberculosis Report 2012). Although notifications remain high, trends in the past five years show a steady stabilization with a slight decline. A total of 11,971 patients were notified in 2012 compared to 13,520 recorded in 2009. The TB burden in Lesotho remains huge. All forms of TB (new and previously treated) have continued to show a linear increase from 1990 onwards with only a slight decline since 2007. Furthermore, TB notification rates have remained above 400 per 100,000 population [3].

Lesotho is listed among the 30 countries with high TB burden. According to the Lesotho Annual Joint Review (AJR) 2017-18 report, treatment success for new and relapse TB patients was 76% among the 2016 TB cohort. This was far below the national target of 90%, and this target was affected by factors such as high death rate, patients not evaluated, and loss of follow-up [5]. In its new National Tuberculosis Strategic Plan 2023-2028, Lesotho adopted two goals; to reduce TB burden by scaling up TB prevention, diagnosis, and care through a people-centered approach; and to strengthen structures and support systems for integrated, efficient, and quality TB services. In order to reach these goals, an effective evaluation of TB treatment outcomes remains the backbone of a successful TB program [6].

**Study aim and objective**: this study aims to evaluate TB treatment outcomes among patients on first-line anti-tuberculosis treatment and identify the factors associated with successful TB treatment outcomes at Senkatana TB clinic in Lesotho from 2025-2017.

**Specific objectives:** i) to determine the proportion of TB patients who experienced treatment success at Senkatana TB Clinic from 2015-2017; ii) to determine predictors for successful TB treatment outcomes of patients registered for anti-tuberculosis treatment first-line regimen at Senkatana TB clinic from 2015-2017.

## Methods

**Study design:** this was a register-based retrospective cohort study of patients on first-line anti-tuberculosis treatment registered at Senkatana TB clinic from 2015 to 2017.

**Study setting:** the study was conducted at Senkatana TB Clinic in Maseru, the capital city of Lesotho. It was established in 2005 and constituted the main referral TB clinic in the country. Tuberculosis services are provided by multidisciplinary teams composed of medical doctors, nurses, pharmacy attendants, counselors, and data clerks.

**Study population:** the study population was all confirmed cases of TB first-line regimen diagnosed at Senkatana TB clinic in Maseru district, Lesotho.

**Inclusion and exclusion criteria:** all patients diagnosed with TB by smear microscopy, Xpert MTB/RIF, and X-ray regardless of history of previous treatment were included in the study. Patients who were not enrolled between January 2015 and December 2017 were excluded from the study as well as MDR TB patients.

**Sampling method:** no sampling method was required as all registered TB patients on first-line regimens were included in the study.

**Data analysis:** a data abstraction tool was designed. Data was captured and cleaned in Epi info version 7, and then exported into Stata version 14 for analysis. Frequency distributions, tables, and graphs were used to describe categorical variables. Bivariate logistic regression analysis was used to determine factors associated with successful TB treatment outcomes. Treatment success was defined as either cured or completed treatment, while poor treatment outcome was either treatment failure, loss to follow-up, or death. The potential factors included socio-demographic characteristics (sex, age, occupation), patient category, mode of referral, and clinical characteristics (type of patient, site of TB, HIV test result, anti-retroviral treatment (ART). A p-value <0.05 was considered statistically significant with odds ratios (OR), 95% confidence intervals (95% CI), and p-values calculated for each predictor.

**Treatment outcome definition:** in this study, treatment outcomes are categorized into successful and unsuccessful treatment outcomes. Successful treatment outcomes included cured and treatment-completed cases. Unsuccessful treatment outcomes included treatment failure cases, defaulters, not evaluated, and patients who died.

### The World Health Organization defines TB treatment outcomes as

**Cured:** a pulmonary TB patient with bacteriologically confirmed TB at the beginning of treatment who was smear- or culture-negative in the last month of treatment and on at least one previous occasion.

**Treatment completed:** a TB patient who completed treatment without evidence of failure but with no record to show that sputum smear or culture results in the last month of treatment and on at least one previous occasion were negative, either because tests were not done or because results are unavailable.

**Treatment failed:** a TB patient whose sputum smear or culture is positive at month 5 or later during treatment.

**Treatment outcome:** the final known status of a TB patient who started anti-TB treatment.

**Lost to follow-up (default):** a TB patient who did not start treatment or whose treatment was interrupted for two consecutive months or more.

**Not evaluated:** a TB patient for whom no treatment outcome is assigned. This includes cases “transferred out” to another treatment unit as well as cases for which the treatment outcome is unknown to the reporting unit.

**Treatment success:** the sum of cured and treatment completed

**Unsuccessful treatment outcome:** the sum of loss to follow-up, death, treatment failure, and not evaluated.

**Ethical consideration and legal consideration:** ethics approval was obtained from the National Health Research Committee of the Lesotho Ministry of Health (ID04-2020) before data collection. Permission was sought from Senkatana ART clinic to gain access to patients´ information. Consent from individual patients was not sought because we used routine data. However, all patient information was anonymized and de-identified before analysis. Access to the database was protected by a password.

## Results

**Demographic characteristics of study participants:** from a total of 1,027 TB patients registered at Senkatana TB Clinic, Maseru, between January 2015 and December 2017, 602 (58.6%) were males, mean standard deviation (SD) age of participants was 39 (12.5) years, and the most patients 215 (20.9%) were 30-34 years of age. Majority of patients 863 (84.0%) had other occupations; these included 492 (57.9%) self-employed and 62 (7.3%) taxi drivers. The next largest high-risk group infected by TB were 102 (9.6%) factory workers. Majority of the TB patients 867 (86.9%) were supported or observed by their family members or friends during treatment. Others constituted occupations not included in the TB register as high-risk groups (Table 1).

**Table 1:** demographic characteristics of the study participants

Variable	2015 (N=392)		2016 (N=376)		2017 (N=259)		Total	
	Frequency	Percentage (%)	Frequency	Percentage (%)	Frequency	Percentage (%)	Frequency	Percentage (%)
**Sex**								
Female	155	39.5	162	43.1	108	41.7	425	41.4
Male	237	60.5	214	56.9	151	58.3	602	58.6
**Age**								
15-19	9	2.3	6	1.6	5	1.9	20	1.9
20-24	21	5.4	20	5.3	10	3.9	51	5.0
25-29	55	14.0	54	14.4	28	10.8	137	13.3
30-34	82	20.9	76	20.2	57	22.0	215	20.9
35-39	64	16.3	63	16.8	61	23.6	188	18.3
40-44	39	9.9	59	15.7	26	10.0	124	12.1
45-49	33	8.4	36	9.6	21	8.1	90	8.8
50-54	39	9.9	26	6.9	14	5.4	79	7.7
55-59	17	4.3	9	2.4	10	3.9	36	3.5
60-64	19	4.8	8	2.1	11	4.2	38	3.7
≥65	14	3.6	19	5.1	16	6.2	49	4.8
<30	1	0.3	3	0.9	0	0.0	4	0.4
31-40	45	13.8	25	7.7	22	9.1	92	10.3
41-50	104	32.0	115	35.6	96	39.7	315	35.4
51-60	127	39.1	107	33.1	74	30.6	308	34.6
61-70	32	9.8	44	13.6	28	11.6	104	11.7
>70	16	4.9	29	9.0	22	9.1	67	7.5
**Occupation**								
Ex-Miner	14	3.6	14	3.7	8	3.1	36	3.5
Factory Worker	31	7.9	56	14.9	15	5.8	102	9.6
Health Worker	4	1.0	4	1.1	2	0.8	10	1.0
Miner	1	0.3	1	0.3	3	1.2	5	0.5
Prisoner	9	2.3	0	0	0	0	9	0.9
Others	333	84.9	301	80.1	229	88.4	863	84.0
**Treatment observer**								
Community health worker (CHW)	12	3.1	10	2.7	27	11.3	49	4.9
Family member (FM) or Friend (FR)	342	87.9	335	90.5	190	79.5	867	86.9
Health Worker (HW)	32	8.2	22	5.9	20	8.4	74	7.4
Traditional healer (TH)	1	0.3	0	0	0	0	1	0.1
Other (Specify)	2	0.5	3	0.8	2	0.8	79	0.7

**Clinical characteristics of the study participants:** out of 1,027 patients, the majority 899 (87.5%) were in category one (CAT I) and category three (CAT III), 868 (84.5%) had pulmonary TB, and 895 (87.1%) were new cases, 93 (9.1%) relapsed, while 24 (2.3%) were retreatment cases (Table 2).

**Table 2:** clinical characteristics of the study participants (N=1027)

Variable	2015 (N=392)	2016 (N=376)	2017 (N=259)	Total	
	n (%)	n (%)	n (%)	Frequency	Percentage (%)
**Treatment category**					
CAT I and III	355(90.6)	323(85.9)	221(85.3)	899	87.5
CAT II	37(9.4)	53(14.1)	37(14.3)	127	12.4
Site					
Pulmonary TB (P)	328(83.7)	325(86.4)	215(83.0)	868	84.5
Extra-pulmonary TB(EP)	64(16.3)	51(13.6)	44(17.0)	159	15.5
**Type of patients**					
New case	348(88.8)	323(85.9)	224(86.5)	895	87.1
Relapse	24(6.1)	38(10.1)	31(12.0)	93	9.1
Treatment after failure (TAF)	3(0.8)	1(0.3)	0	4	0.4
Treatment after loss to follow-up (LTF)	3(0.8)	4(1.1)	3(1.2)	10	1.0
Transfer In	1(0.3)	0	0	1	0.1
Other previously treat	13(3.3)	10(2.7)	1(0.4)	24	2.3
**TB treatment outcomes**					
Cure	99(25.3)	48(12.8)	52(20.1)	199	19.4
Treatment complete	189(48.2)	228(60.6)	138(53.3)	555	54.0
Died	39(9.9)	47(12.5)	32(12.4)	118	11.5
Lost to follow up	15(3.8)	15(4.0)	17(6.6)	47	4.6
Treatment failure	1(0.3)	1(0.3)	3(1.2)	5	0.5
Not evaluated	49(12.5)	37(9.8)	17(6.6)	103	10.0
TB treatment success	288(73.5)	276(73.4)	190(73.4)	754	73.4

TB: tuberculosis

**TB/HIV characteristics of the study participants:** a total of 843 (82.1%) of the patients were co-infected with HIV, of which 778 (92.3%) were on ART and 839 (99.9%) were on Co-trimoxazole (Table 3).

**Table 3:** TB/HIV characteristics of the study participants

Variable	2015 (n=311)	2016 (n=322)	2017 (n=210)	Total	
	N (%)	N (%)	N (%)	Frequency	Percentage (%)
**HIV test result**					
Positive	311 (79.3)	322 (85.6)	210 (81.1)	843	82.1
Negative	68 (17.3)	44 (11.7)	49 (18.9)	161	15.7
Missing result	13 (3.3)	10 (2.7)	0	23	2.2
**Antiretroviral treatment**					
On ART	278 (89.4)	293 (91.0)	207 (98.6)	778	92.3
Not on ART	33 (10.6)	27 (8.4)	3 (1.4)	63	7.5
Missing	0	2 (0.6)	0	2	0.2
**Prevention opportunistic infection**					
On co-trimoxazole	311 (99.7)	321 (100.0)	207 (100)	839	99.9
Depsone	1 (0.3)	0	0	1	0.1

TB: tuberculosis

**Figure 1:**
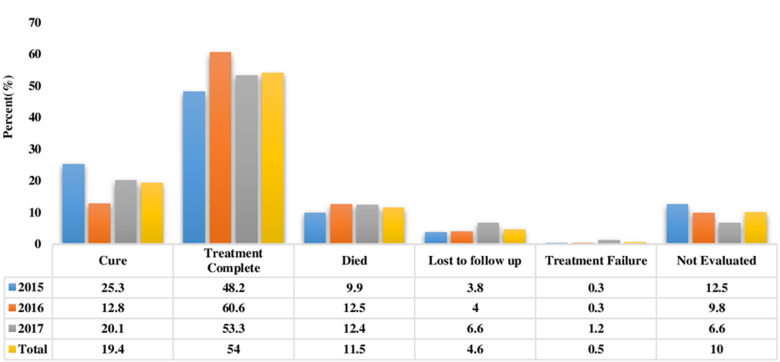
tuberculosis treatment outcomes at Senkatana 2015-2017

**Figure 2:**
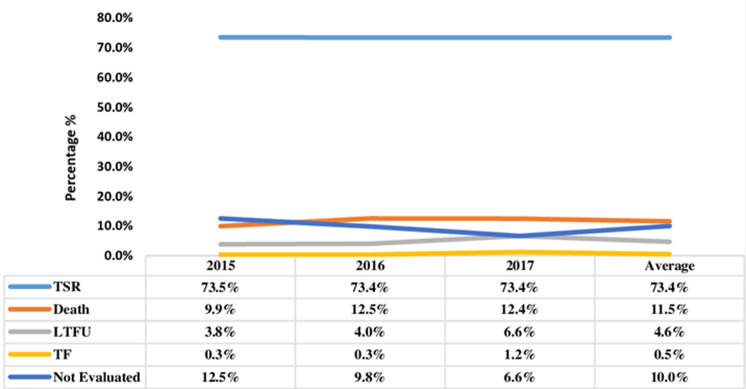
treads in treatment success rate (TSR) and other unsuccessful outcomes at Senkatana 2015-2017

**Treatment outcomes and trend in TB treatment success rate:** out of the 1,027 TB patients, 199 (19.4%) were cured, 555 (54.0%) completed treatment, 118 (11.5%) died, 47 (4.6%) were lost to follow-up (LTFU), 5 (0.5%) had treatment failure and 103 (10.0%) were not evaluated (Figure 1). Overall, 754 (73.4%) had successful TB treatment outcomes and treatment success remained at 73%, which is below the WHO target of 90%, during the study period from 2015 to 2017. On the other hand, the case fatality rate remained above the WHO target of 5% during the same period (Figure 2).

### TB treatment outcomes

**Figure 3:**
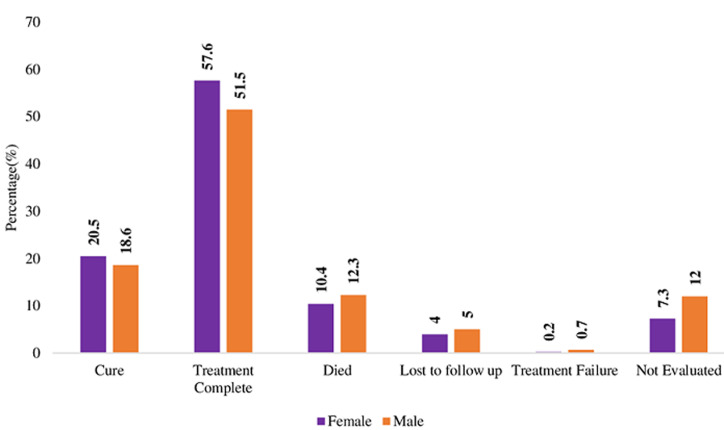
treatment outcomes and sex (female: n= 425, male: n=602)

**Figure 4:**
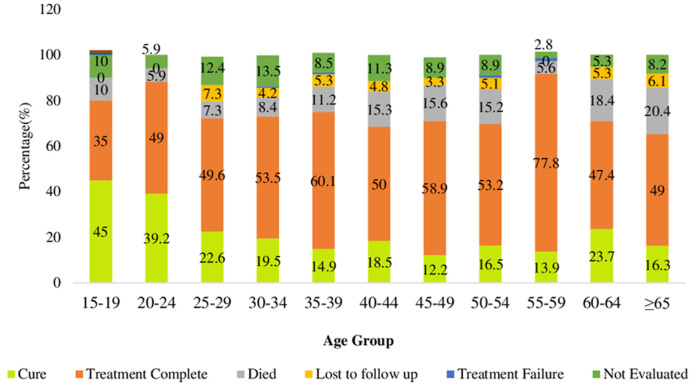
treatment outcomes by age group

**Description of treatment outcomes by demographic characteristics:** there were slightly more females than males who were cured (20.5% vs 18.6%) and who completed treatment (57.6% versus 51.5%). On the other hand, slightly more males than females died from the disease (12.3% vs 10.4%), were LTFU (5% vs 4%) or had treatment failure (0.7% vs 0.2%) (Figure 3). In terms of age, adolescents (15-19 years) had the best cure rate (45%) than any other age group, followed by young people (20-24 years) at 39.2%. Adults aged 55-59 years had the highest treatment completion rate (77.8%) than any other age group, followed by 35-39 years age group (60.1%). The oldest age group of 65 years and above had the highest mortality (20.4%) than any other age group, followed by 60-64 years age group (18.4%). LTFU was highest among young adults aged 25-29 years (7.3%) and lowest among 45-49 years age group (3.3%) (Figure 4).

**Description of treatment outcomes by clinical characteristics:** the completion rate was better for patients who were previously treated for TB at 66.7% (n=24) followed by treatment after loss to follow-up (LTFU) at 60.0% (n=10). Death rate was high among both new and relapse cases at around 12%. Generally, mortality among TB is still high across type of patients. In this study, LTFU was high among treatment after LTFU and previously treated patients (PTB). For site of TB, cure rate was better among pulmonary tuberculosis patients than extra pulmonary tuberculosis patients (EPTB) while the completion rate was better for EPTB patients. Death rate was almost the same for PTB and EPTB patients at around 13%. Cure rate was better in HIV-negative patients at 33.5% (n=161) while the completion rate was good in TB/HIV co-infected patients at 57.2% (n=843). Tuberculosis/HIV co-infected patients had higher mortality than HIV-negative TB patients. Not evaluated, mortality, and LTFU were highest among patients with missing results for HIV at 43.5%, 34.8%, and 13% (n=23), respectively. Treatment success rate was high among HIV co-infected TB patients who were taking ART during TB treatment at 78.6% (n=778) than those not taking ART at 23.8% (n=63). The death rate was higher among HIV co-infected TB patients who were not taking ART during TB treatment at 46% (n=63) than those taking ART at 8.5% (n=778). Not evaluated and LTFU was highest among patients with HIV co-infected TB patients who were not taking ART during TB treatment at 22.2% and 7.9%, respectively (Table 4).

**Table 4:** treatment outcomes and clinical variables

Variables	TB Treatment outcomes (n=1027)						Total
	Cure	Treatment Complete	Died	Lost to follow up	Treatment Failure	Not Evaluated	
**Type of Patient**							
New case	180(20.1)	482(53.9)	104(11.6)	35(3.9)	4(0.4)	90(10.1)	895
Relapse	15(16.1)	50(53.8)	11(11.8)	8(8.6)	1(1.1)	8(8.6)	93
Treatment after failure (TAF)	3(75.0)	0	0		0	1(25.0)	4
Treatment after loss to Follow-up (LTF)	1(10.0)	6(60.0)	1(10.0)	1(10.0)	0	1(10.0)	10
Transfer In	0	1(100)	0	0	0	0	1
Other Previously Treat	0	16(66.7)	2(8.3)	3(12.5)	0	3(12.5)	24
**Site**							
Pulmonary TB (P)	189(21.8)	450(51.8)	98(11.3)	42(4.8)	4(0.5)	85(9.8)	868
Extra-Pulmonary TB (EP)	10(6.3)	105(66.0)	20(12.6)	5(3.1)	1(0.6)	18(11.3)	159
**HIV Test result**							
Positive	144(17.1)	482(57.2)	97(11.5)	38(4.5)	3(0.4)	79(9.4)	843
Negative	54(33.5)	72(44.7)	13(8.1)	6(3.7)	2(1.2)	14(8.7)	161
Missing result	1(4.3)	1(4.3)	8(34.8)	3(13.0)	0	10(43.5)	23
**Antiretroviral Treatment**							
On ART	139(17.9)	472(60.7)	66(8.5)	33(4.2)	3(0.4)	65(8.4)	778
Not on ART	5(7.9)	10(15.9)	29(46.0)	5(7.9)	0	14(22.2)	63
Missing	0	0	2(100)	0	0	0	2

**Demographic and clinical characteristics associated with successful TB treatment outcomes:** in unadjusted (univariate) analyses, age, sex, and ART were found to be significantly associated with successful TB treatment outcomes among TB patients. The odds of successful TB treatment outcome were higher in females than males (78.1% vs 70.1%, OR 1.52, 95%CI: 1.14 - 2.03, p=0.004). With regards to age, the odds of successful TB treatment outcome were higher for the 20-24 years age group (88.2% vs 65.3%, OR 3.98, 95% CI: 1.42 - 11.22, p=0.009) and 55-59 years (91.7% vs 65.3%, OR 5.84, 95% CI: 1.56 - 21.88, p=0.009), compared to ≥ 65 years age group. The odds of successful TB treatment outcomes were higher among HIV co-infected TB patients who were taking ART during TB treatment than those not taking ART (75.8% vs 23.8%, OR 11.70, 95% CI: 6.40 - 21.43, p<0.001). There was no significant statistical difference in the odds of successful TB treatment outcomes when comparing new versus relapse patients (p=0.397), pulmonary versus extra-pulmonary TB patients (p=0.735), and HIV infected versus HIV uninfected TB patients (p=0.284) (Table 5). Patients observed by family members or friends were more likely to achieve treatment success (p=0.014) (Table 6).

**Table 5:** demographic and clinical characteristics associated with successful TB treatment outcomes

Variables	Treatment Outcome		Total	OR (95% CI)	P-value	X2	X2 P-value
	Successful	Unsuccessful					
**Sex**							
Female	332(78.1)	93(21.9)	425	1.52(1.14 - 2.03)	0.004*	8.2	0.004*
Male	422(70.1)	180(29.9)	602	Ref	-		
**Age**							
15-19	16(80.0)	4(20.0)	20	2.13(0.61-7.37)	0.235	19.2	0.038
20-24	45(88.2)	6(11.8)	51	3.98(1.42-11.22)	0.009*		
25-29	99(72.3)	38(27.7)	137	1.38(0.69-2.78)	0.361		
30-34	157(73.0)	58(27.0)	215	1.44(0.74-2.78)	0.281		
35-39	141(75.0)	47(25.0)	188	1.59(0.81-3.13)	0.176		
40-44	85(68.5)	39(31.5)	124	1.16(0.58-2.33)	0.681		
45-49	64(71.1)	26(28.9)	90	1.31(0.62-2.75)	0.479		
50-54	55(69.6)	24(30.4)	79	1.22(0.57-2.60)	0.611		
55-59	33(91.7)	3(8.3)	36	5.84(1.56-21.88)	0.009*		
60-64	27(71.1)	11(28.9)	38	1.30(0.52-3.26)	0.570		
65 and above	32 (65.3)	17 (34.7)	59	Ref	_		
**Type of Patient**							
New case	662(74.0)	233(26.0)	895	1.22(0.77-1.95)	0.397	0.72	0.396
Relapse	65(69.9)	28(30.1)	93	Ref			
**Site of TB**							
Pulmonary TB (P)	639(73.6)	229(26.4)	868	1.07(0.74-1.55)	0.735	0.11	0.735
Extra-Pulmonary TB (EP)	115(72.3)	44(27.7)	159	Ref	-		
**HIV Test result**							
Positive	626(74.3)	217(25.7)	843	0.80(0.53-1.20)	0.284	1.15	0.283
Negative	126(78.3)	35(21.7)	161				
**Antiretroviral Treatment**							
On ART	611(78.5)	167(21.5)	778	1.60(1.12-2.29)	0.001*	91.7	0.001*
Not on ART	15(23.8)	48(76.2)	63	11.70(6.40-21.43)	0.000*		
**Occupation (High risk group)**							
Factory worker	84 (82.4)	18 (17.6)	102	1.77 (1.04 - 3.01)	0.034*	4.59	0.032*
Other occupation	669(72.5)	254(27.5)	923	Ref			

Statistically significant association at p-value of < 0.05

**Table 6:** factors associated with successful TB treatment outcome: results from the multivariate logistic regression analysis (n = 1027)

Variable	aOR (95%CI)	P-value
**Antiretroviral treatment (ART)**		
On ART	12.87 (6.86 - 24.13)	< 0.001
Not on ART	Ref	
**Treatment observer**		
Family member or friend	1.87 (1.13 - 3.08)	0.014
Health worker	Ref	
**Sex of the patient**		
Female	1.49 (1.05 - 2.13)	0.026
**Male**	**Ref**	

## Discussion

The current study provides information about treatment outcomes of TB and associated factors of patients registered at Senkatana TB clinic in Lesotho from 2015 to 2017. In the present study, a total of 107 patients were registered, of which most (58.6%) were male. This finding was consistent with previous studies conducted in Pakistan [7] and Zambia [8]. The high proportion of males in our study may be due to the consistency with the epidemiology of TB, as males are more exposed to infection in the community than females [9]. This study also found that majority of patients were from the economically productive age group of the society (30-44) years. Similar findings were reported in studies conducted in Ethiopia [10-12]. This finding indicates that TB affected mainly the economically productive age group of the society, which can affect the economy of developing countries like Lesotho. It was also found that the majority of the study subjects (84.5%) presented with pulmonary TB. This finding was comparable with studies from Nigeria [13] and Pakistan [7]. This may be due to the fact that extra-pulmonary TB is diagnosed late than pulmonary TB. The study further found out that the proportion of HIV co-infected patients with pulmonary TB was higher than the proportion of co-infected patients with extra-pulmonary TB (EPTB). However, this difference was not statistically significant. This is in contrast with the study conducted in Ghana [14] where HIV positivity was found to be significantly associated with EPTB compared with PTB.

Treatment success rate in our study was 73.4%, which was still below the WHO target of 85%. Furthermore, TB treatment success rate was low and constant over the three years period. This finding was slightly lower than the national TB treatment success rates of the 2016 cohort (76%) and the rate recommended by WHO [4]. This finding was lower than findings of the studies conducted in Ethiopia of 85.2% [11]. Treatment outcome of tuberculosis patients under directly observed treatment short course and factors affecting outcome in southern Ethiopia: a five-year retrospective study and 91.5% [15]. In contrast, the treatment success rate observed in this study was higher than the results reported from studies conducted in Nigeria (56.5%) [13] and South Africa (67%) [16]. However, it is lower than the national average reported in 2016 cohort. Our study revealed that age groups of 20-24 and 55-59 years, female, and negative HIV status were associated with successful TB treatment outcomes. This finding is in accordance with a similar study carried out in Nigeria [17] where patients aged 14 years and above were 12 times more likely to have successful TB treatment outcomes. With regards to female gender, studies conducted in Nigeria [18] and Afghanistan [19] also found that female patients had better TB treatment outcomes than male patients. Lastly, HIV-negative TB patients (78.2%) were more likely to have a better treatment outcome as compared to HIV-co-infected TB patients (74.3%), and this finding is observed by other studies [15,20]. The lower successful TB treatment in HIV co-infected patients can be attributed to the fact that as HIV infection progresses, CD4 cell count declines by about 50-80 cells/mm^3^ per year and the overall immune system of the person becomes less able to prevent the dissemination of M. tuberculosis in the body [21].

This study reported treatment failure, death, not evaluated, and loss to follow-up rates of 0.5%, 11.5%, 10%, and 4.6%, respectively. These constituted an overall unsuccessful TB treatment outcome rate of 26.6%, which was higher than the 10.8% unsuccessful rate reported in Tigray region [22]. The lower treatment failure rate observed in this study may be due to good treatment adherence and the presence of village healthcare workers in the community. A similar low rate has been reported in Ethiopia (0.3%) [11], Malaysia (1%) [23], Afghanistan (3.5%) [24], and in India (1.6%) [25]. The death rate reported in this study (11.5%) is almost similar to the rate observed in Ghana of 10.2% [26]. In contrast, studies conducted in Ethiopia [11], Afghanistan [24], and Somalia [20] found a low death rate of 1%, 0.5%, and 2.3%, respectively. Our finding is slightly lower than the national TB death rate of 13% among the 2016 cohort and above the WHO target of 5%. Mortality among TB patients was high between the age group of 25-54 years and age 60 years and above. This corresponds to findings of studies conducted in Finland [27] and Brazil [28], where increasing age was strongly associated with death. Older age has been reported to be a risk factor for death due to lowered immunity and co-morbidities [29].

These findings highlight the importance of providing close follow-up for older age patients to increase their successful TB treatment outcomes. Furthermore, the present study notes high death rate (46.5%) among HIV co-infected TB patients who were not taking ART during TB treatment, with similar observations have been reported in Uganda [30]. With regard to HIV status, HIV co-infected TB patients had a higher death rate compared to HIV-negative TB patients. This is in agreement with a study conducted in Nigeria [31]. High death rate was also found among TB patients who missed HIV test results and this was in agreement with a study conducted in Cameroon [32], where failure to undergo HIV testing was an independent risk factor of death. High death rate in our study might be due to lack of follow-up or late referral of TB patients to Senkatana TB clinic. The rate of patients not evaluated (10%) in our study was higher than the rate observed in the National TB report 2016 cohort and above WHO target of 3% [7]. Contrary to our finding, a study in Zambia found a high rate of patients not evaluated at 29% [8]. Furthermore, more males were not evaluated compared to females at 12% and 7.3%, respectively. The high rate of patients not evaluated in our study could be due to many factors, including poor record keeping in health facilities, immigration special for male patients, and shortage of human resources. The loss to follow-up rate in this study of 4.6% was slightly lower compared to the rate reported in Ethiopia of 5.3% [33] and was comparable with the rate observed in the national report 2016 cohort (4%). Contrary to our findings, studies conducted in Yemen [33] and Nigeria [13] reported higher rates of patient loss to follow-up at 12.6% and 17.4%, respectively. With regard to HIV status, loss to follow-up rate was slightly higher among HIV co-infected TB patients than HIV-negative patients with 4.5% and 3.7%, respectively. This was in agreement with a study conducted in Nigeria [31].

This study also revealed a high loss to follow-up rate among miners (40%) and this may be due to migration as most miners are working in South Africa. Furthermore, loss to follow-up was highest among young adults aged 25-29 years and lowest among aged 45-49 years. High loss to follow in our study may be due to poor mechanisms of loss to follow-up tracing, poor health education, or higher pill burden due to TB/ART co-treatment and social stigma. The current study has strengths as well as limitations which need to be noted while interpreting the findings. The main strength of the study is that it was conducted at a main referral TB clinic, hence the findings reflect the treatment outcome of health centers in the country. In terms of limitations, the study used routinely collected data that may have been subject to reporting errors and data quality depending on the TB program. Furthermore, there are variables that can affect the TB treatment outcome that were not included in the TB register (e.g. marital status, education, nutrition, distance from the TB clinic, risk factors, location, monthly income, and others).

## Conclusion

This study showed that TB treatment success rate was low and constant over the period of three years. Death rate, loss to follow-up, and non-evaluation were high among our study participants. HIV co-infected TB patients had higher death rate compared to HIV-negative TB patients with a higher death rate among TB patients who missed HIV test results as well as among HIV co-infected TB patients who were not taking ART during TB treatment. Based on these findings, close follow-up should be placed on improving TB treatment outcomes among HIV co-infected and male patients. In addition, we recommend proper recording of data.

### What is known about this topic

- HIV co-infection in TB patients is associated with unfavourable treatment outcomes;
- TB is a major public health problem and the second leading cause of mortality globally from an infectious agent;
- United Nations through the WHO End TB Strategy committed to 90% reduction in TB deaths and an 80% reduction in the TB incidence rate by year 2030.

### What this study adds

- TB treatment success rate was low and constant over the period of three years;
- The age groups of 20-24 and 55-59 years were more likely to have better cure rates and completion rates respectively than any other age group; male patients were less likely to have high completion rates than female patients;
- Death rates, loss to follow and not evaluated rates were high among our study participants and above the WHO targets; it is important to evaluate all the patients.
